# Linking spontaneous speech, cognition, and psychopathology across affective and psychotic disorders: A network approach

**DOI:** 10.1192/j.eurpsy.2026.10151

**Published:** 2026-01-23

**Authors:** Rieke Roxanne Mülfarth, Svenja Seuffert, Nina Alexander, Hamidreza Jamalabadi, Igor Nenadić, Benjamin Straube, Lea Teutenberg, Florian Thomas-Odenthal, Paula Usemann, Udo Dannlowski, Tilo Kircher, Frederike Stein

**Affiliations:** 1 Faculty of Medicine, Department of Psychiatry and Psychotherapy, Philipps-Universität Marburg, Marburg, Germany; 2 Center for Mind, Brain and Behavior, Philipps-Universität Marburg, Marburg, Germany; 3 Institute for Translational Psychiatry, University of Münster, Münster, Germany; 4 Department of Psychiatry, Medical School and University Medical Center OWL, Protestant Hospital of the Bethel Foundation, Bielefeld University

**Keywords:** cognition, natural language processing, network analysis, psychopathology, transdiagnostic

## Abstract

**Background:**

Language impairments are common in affective and psychotic disorders, yet their patterns and underlying pathomechanisms remain insufficiently understood. A transdiagnostic perspective provides a framework for identifying shared and disorder-specific language alterations across diagnostic boundaries. Combining natural language processing (NLP) with network analysis enables the investigation of complex associations between linguistic, cognitive, and psychopathological features.

**Methods:**

Spontaneous speech from *N* = 372 participants (119 MDD, 27 BD, 48 SSD and 178 HC) was elicited using four Thematic Apperception Test pictures (~12 min per participant). NLP models were applied to extract latent linguistic variables across various levels, including lexical diversity, syntactic complexity, semantic coherence, and disfluencies. Network analysis was used to relate linguistic variables, psychopathology (SAPS, SANS, HAM-A, HAM-D, YMRS, TLI, GAF), and cognitive performance (attention, verbal memory, recognition, and verbal fluency).

**Results:**

Linguistic variables formed the densest network cluster, with type–token ratio, mean length of utterance, and syntactic complexity emerging as central nodes. Psychopathology variables were less cohesive, while TLI “Impoverishment”, coherence mean, and executive functioning bridged linguistic, cognitive, and psychopathological domains. Network comparison tests revealed no significant differences in linguistic–cognitive network structure across HC, MDD, BD, and SSD.

**Conclusions:**

Linguistic networks show high structural consistency across healthy individuals and patients, whereas psychopathological symptom networks reflect transdiagnostic profiles. These findings support a dimensional and transdiagnostic framework underscore shared language–cognition mechanisms, and highlight executive functioning as key cross-domain connection, which opens up new avenues for dimensional research into the pathophysiological and etiological mechanisms underlying language dysfunctions.

## Introduction

Language disturbances are increasingly understood as complex phenomena that cut across traditional diagnostic boundaries, affecting both affective disorders (e.g., major depressive disorder (MDD), bipolar disorder (BD)) and psychotic disorders (e.g., schizophrenia (SZ), schizoaffective disorder (SZA) – collectively referred to as schizophrenia spectrum disorders (SSD)). Beyond clinical symptoms, language impairments are associated with social withdrawal, reduced life satisfaction, and poorer functional outcomes [1]. Formal thought disorder (FTD), referring to impairments in thought and language, has been shown to negatively impact psychosocial quality of life, highlighting the need to address cognitive–linguistic deficits [2]. Similarly, even when clinically stable, patients report social functioning impairment, underscoring the importance of identifying barriers to full psychosocial recovery [3]. FTD includes positive (e.g., disorganization) and negative (e.g., poverty of speech) dimensions. It is noteworthy that the prevalence of FTD is often overlooked in MDD, albeit with prevalence rates up to 53%. In SZ and BD, rates reach up to 81%, while in SZA up to 60%. Moreover, FTD likely exists on a continuum with normal speech, as evidence of it has been observed in 6% of healthy controls (HC) [4].

FTD has been the most studied in SSD, typically manifesting as derailment, incoherence, or neologisms but also as reduced speech production with increased response latencies and blocking [4–7]. In MDD, findings point out to increased response latencies, diminished verbal output, and a tendency toward negatively valenced language [5, 8]. Across disorders, FTD severity has been linked to relapse, (re-)hospitalization, and impaired psychosocial functioning [4, 9]. Recent studies emphasize the transdiagnostic nature of FTD. Stein et al. [8] have identified a three-factor model (disorganization, emptiness, and incoherence) across affective and psychotic disorders, replicated by Tang et al. [10], who used different FTD rating scales and patients. A further study applying different clustering algorithms in recent onset psychosis (both affective and psychotic) showed two FTD subtypes, i.e., high FTD versus low FTD [10]. Most recently, this approach was expanded to a large sample of affective and psychotic disorders across different illness stages. Using a model-based cluster analytic approach, four subtypes ranging from minimal to severe FTD were identified with clinical diagnoses to be distributed across all of them [11].

Given the prevalence of FTD across diagnoses and the growing evidence of its transdiagnostic nature, it becomes clear that studying FTD and speech disturbances beyond traditional diagnostic categories is essential for a deeper understanding of their association with cognition, communication, and daily functioning. By focusing on shared mechanisms rather than on disorder-specific symptoms, a transdiagnostic perspective facilitates the identification of universal and overlapping language-related alterations. This approach is particularly advantageous in addressing comorbidity, which is a prevalent challenge in psychiatry. Traditional diagnostic boundaries, such as those defined by the Diagnostic and Statistical Manual of Mental Disorders (DSM; [13]) or the International Classification of Diseases (ICD; [14]), often fail to capture the shared mechanisms underlying different disorders [12, 13]. For example, dysregulated cognitive and emotional processes, including rumination, impaired executive function, and semantic network disturbances, are common across disorders like SSD, MDD, and BD [8, 12, 14–19].

Traditional FTD assessments, such as the Scale for the Assessment of Thought, Language, and Communication (TLC; [20]), the Scale for the Assessment of Objective and Subjective Formal Thought and Language Disorder (TALD; [21]), and the Scale for the Assessment of Positive Symptoms (SAPS; [25]), are clinically useful but limited by scalability and subjectivity [22]. Therefore, natural language processing (NLP) provides an advanced and highly sensitive methodology for detecting and analyzing language impairments across multiple linguistic domains. By facilitating an objective, scalable, and systematic assessment of linguistic features, including syntax, semantics, and morphology, NLP offers a powerful tool for uncovering subtle language abnormalities [23–28]. Applying NLP-based methods to spoken language, studies have predominantly focused on SSD patients [25], with only some examining MDD [29]. In addition, only a few have investigated NLP metrics across multiple psychiatric disorders at the same time [30, 31]. In SSD, syntax-focused measures, such as reduced clause complexity and diminished syntactic diversity, have been linked to working memory deficits and impaired executive functioning [26, 32–36]. In MDD, computational methods have revealed increased usage of first-person pronouns and negative affective language, reflecting heightened self-focus and cognitive distortions [37–40].

Nevertheless, the interrelationship between different NLP-derived metrices and other domains, including cognition and psychopathology, remains poorly understood [22]. In this context, network analysis offers a powerful framework to gain a more comprehensive understanding of the complex architecture of both distinct and shared underlying mechanisms. Unlike traditional linear models, network approaches enable both the visualization and quantification of complex, multidimensional interactions [41]. In the context of NLP, network analysis serves as a powerful complementary tool, particularly for modeling the intricate dependencies between linguistic patterns, psychopathological features, and cognitive dysfunctions. This synergy enables the identification of meaningful associations that might otherwise remain undetected when using standard linguistic feature extraction or classification methods alone. In psychosis research, network methods have been used to analyze semantic and syntactic disruptions and their links to symptoms [1, 34]. By analyzing spontaneous speech from SSD, MDD, and HC using syntactic measures and network analyses, Schneider et al. [30] found that SSD patients exhibit significantly reduced syntactic complexity and diversity. Lower complexity was associated with more severe symptoms and cognitive deficits, while network analyses highlighted distinct roles for complexity and diversity in language processing [30]; however, this study extracted syntactic features manually instead of using NLP. Prior research has also linked semantic network alterations to the severity of thought disorder symptoms in psychosis. Nettekoven et al. constructed semantic speech networks from transcribed language, showing that their structure correlates with disorganized thinking [1].

Previous studies often applied either NLP or network analysis in isolation. Notably, Chavez-Baldini et al. used a transdiagnostic network approach to examine symptom interactions over time, identifying central roles for self-perception and suicidal ideation [17, 42]. Combining the methods of NLP and network analysis in a transdiagnostic perspective is rare [43]. More recently, Arribas et al. combined NLP and temporal network analysis to study prodromal symptoms across disorders, showing highly interconnected networks with minor differences between diagnoses [43].

Despite these advances, the field remains limited by small samples, manual linguistic annotation (e.g., [30]), and diagnosis-specific designs. To address these gaps, this study integrates NLP and network analysis in a transdiagnostic framework and investigates latent linguistic variables in spoken language across individuals with SSD, MDD, BD, and HC within a German-speaking sample. Specifically, we aim to (1) extract linguistic features on various linguistic levels in spontaneous speech, (2) compare spontaneous speech performance across diagnostic and HC groups, (3) explore the relationship between language, language-related cognition, and psychopathology, and (4) identify domain-specific and cross-domain connections in a transdiagnostic network analysis approach. To explore interplay patterns, analyses included 28 linguistic variables (covering numerous linguistic levels), 10 psychopathological variables, and 6 cognitive function variables. Examining their associations is crucial, as these domains are intricately interconnected, with language serving as both an expression and diagnostic tool for cognitive and psychological processes, while cognitive functions influence linguistic abilities and psychopathological states [44–46].

## Methods

### Participants

We included *N* = 372 German-speaking participants (*n* = 119 MDD, *n* = 27 BD, *n* = 48 SSD, and *n* = 178 HC) (cf. Table 1) from the FOR2107 cohort (see [5], www.for2107.de). Participants were aged between 18 and 67 years and fluent in German. Patients were recruited from the university hospital in Marburg and healthy controls via public advertisement. During a semi-structured clinical interview, diagnoses were acquired according to DSM-IV-TR Axis I disorders (SCID-I; [47]). Trained staff assessed multiple psychopathological rating scales. The patient sample covered a broad range of illness phases, from acute to chronic to remitted states. Exclusion criteria for all participants included neurological or serious medical conditions, verbal IQ <80, and current substance abuse or dependence, or benzodiazepine intake. All participants provided written informed consent, and procedures were approved by the local ethics committees in compliance with the Declaration of Helsinki.

**Table 1.** Sample characteristics, *N* = 372; *n* = 178 healthy controls and *n* = 194 patients

	Major depressive disorder(*n* = 119)	Bipolar disorder(*n* = 27)	Schizophrenia spectrum disorders(*n* = 48)	Healthy controls (*n* = 178)	Group comparison (H/F)	Effect size
Age	44.26 (13.86)	44.55 (12.15)	41.17 (12.20)	44.36 (13.47)	*p* = .472 (H = 2.51)	*η^2^* = .006
Sex	f = 74 m = 45	f = 17 m = 10	f = 18 m = 30	f = 114 m = 64	*p* = .008 (χ^2^ = 11.66)	*V* = .177
Education (in years)	13.40 (2.99)	13.40 (3.06)	12.02 (2.15)	14.56 (3.17)	***p* < .001ᵃ** (H = 27.08)	*η^2^* = .076
Verbal IQ	116.18 (14.21)	111.8 (13.18)	111.89 (15.14)	116.06 (12.83)	*p* = .276 (H = 3.86)	*η^2^* = .015
Age of onset	28.48 (13.82)	24.34 (11.38)	18.79 (11.38)	-	*p* = 1.00 (H = 12.97)	*η^2^* = .074
Psychopathology						
SANS sum	7.11 (7.59)	9.32 (9.78)	18.13 (12.78)	1.33 (2.81)	***p* < .001ᵇᶜᵈ** (H = 135.48)	*η^2^* = .384
SANS nFTD	1.07 (1.89)	1.48 (2.57)	2.70 (2.91)	0.25 (0.75)	***p* < .001**ᵃᶜ**ᵉ** (H = 68.09)	*η^2^* = .181
SAPS pFTD	1.24 (2.55)	4.48 (6.55)	6.09 (7.62)	0.54 (1.52)	***p* < .001**ʰᴷ (H = 64.65)	*η^2^* = .217
SAPS sum	1.45 (2.83)	6.00 (9.03)	13.16 (13.80)	0.53 (1.28)	***p* < .001**ᵃᶜᵈᶠ (H = 99.49)	*η^2^* = .346
HAM-D sum	5.66 (5.79)	5.81 (5.82)	9.24 (8.4)	1.33 (2.16)	***p* < .001**ᵇᶜ (H = 115.44)	*η^2^* = .249
HAM-A sum	7.61 (6.88)	9.37 (9.14)	11.00 (10.05)	1.92 (2.80)	***p* < .001**ᵇ (H = 108.75)	*η^2^* = .252
YMRS sum	.43 (1.00)	5.81 (8.85)	6.00 (7.16)	0.40 (1.25)	***p* < .001**ʰᴷ (H = 97.87)	*η^2^* = .272
GAF	67.71 (12.47)	59.70 (13.25)	51.91 (15.65)	85.36 (9.01)	***p* < .001**ᵇʲ (H = 187.69)	*η^2^* = .536
TLI Impoverished	0.54 (1.08)	0.58 (.73)	0.69 (1.11)	0.33 (.57)	*p* = .040 (H = 8.31)	*η^2^* = .023
TLI Disorganization	1.05 (1.24)	0.91 (1.23)	0.79 (.94)	0.59 (.80)	*p* = .010 (H = 11.27)	*η^2^* = .037
Cognition						
Semantic VF	23.46 (5.32)	20.30 (5.67)	18.45 (5.66)	23.39 (4.96)	***p* < .001**ᵃʲ (F = 13.61)	*η^2^* = .104
Lexical VF	10.99 (4.31)	8.96 (3.32)	9.19 (4.16)	11.78 (4.14)	***p* = .001** (F = 6.98)	*η^2^* = .056
Alternating VF	14.88 (3.38)	13.11 (3.52)	12.08 (3.88)	15.99 (3.15)	***p* < .001**ᵍʲ (H = 44.90)	*η^2^* = .140
Verbal episodic memory (VLMT)	57.15 (10.25)	50.04 (11.34)	46.56 (9.75)	58.48 (9.19)	***p* < .001**ᵍʲ (H = 52.39)	*η^2^* = .154
Recognition (VLMT)	13.26 (2.82)	11.48 (3.40)	10.25 (4.14)	13.49 (2.15)	***p* < .001**ᵃʲ (H = 45.98)	*η^2^* = .137
Executive functioning (TMT_diff)	28.96 (21.17)	48.41 (28.92)	37.83 (26.19)	25.20 (14.72)	***p* < .001**ᶠⁱ (H = 24.79)	*η^2^* = .098

*Note:* Means (standard deviation); *SANS* scale for the assessment of negative symptoms [51], *SAPS* scale for the assessment of positive symptoms [52], *HAM-D* Hamilton rating scale for depression [48], *HAMA* Hamilton anxiety rating scale [49], *YMRS* Young mania rating scale [50], *GAF* global assessment of functioning [47], *TLI* Thought and Language Index [58], *VF* verbal fluency (RWT; [56]), *VLMT* Verbal Learning and Memory Test [54], *TMT* Trail Making Test [55]. *F* = F-statistic from one-way ANOVAs; *H* = H-statistic from Kruskal–Wallis tests (used when ANOVA assumptions were not met); *η^2^* = Eta-squared, an effect size measure for ANOVA, indicating the proportion of variance explained by group differences; *V* = Cramér’s *V*, an effect size measure for chi-squared tests, reflecting the strength of association between categorical variables.Post hoc correction for multiple testing (Bonferroni) with significant results **in bold font**.

ᵃ = HC > SSD. ᵇ = HC > MDD, BD, SSD. ᶜ = MDD < SSD. ᵈ = BD < SSD. ᵉ = HC > MDD. ᶠ = HC < BD. ᵍ = HC > BD, SSD. ʰ = MDD < BD, SSD. ⁱ = MDD < BD. ʲ = MDD > SSD. ᴷ = HC < BD, SSD.

### Psychopathology and cognition assessment

Following the diagnostic assessment described above, symptom severity was assessed using the Hamilton Rating Scale for Depression (HAM-D; [48]), Hamilton Anxiety Rating Scale (HAM-A; [49]), Young Mania Rating Scale (YMRS; [50]), and the Scales for the Assessment of Negative and Positive Symptoms (SANS; [51], SAPS; [52]), including FTD subscales (i.e., positive FTD and alogia). Overall functioning was assessed via the Global Assessment of Functioning (GAF; [47]). All interviewers were trained and had expertise in administering and scoring the rating scales. Interrater reliability was evaluated using the interclass correlation coefficient, yielding good average reliability of r > .86 across all scales. Cognitive testing focused on language-related measures [53], including verbal episodic memory (VLMT; retrieval and recognition [54]). Executive functioning was assessed using the Trail Making Test (TMT; [55]), and verbal fluency using the Regensburger verbal fluency test (RWT; [56]) across three conditions: semantic (category ‘animals’), lexical (initial letter ‘p’), and alternating verbal fluency (alternating categories ‘fruits’ and ‘sports’).

### Speech data assessment

Spontaneous speech was elicited using four Thematic Apperception Test pictures [57], with three-minute storytelling per picture (~12 min speech). Participants were encouraged to elaborate using nondirective prompts. Speech was transcribed verbatim and preprocessed (e.g., removal of interviewer speech, timestamps, and special characters). Additionally, trained raters who were blind to diagnosis evaluated speech using the Thought and Language Index (TLI; [58]). For each picture and minute, eight items were rated: (i) poverty of speech; (ii) weakening of goal; (iii) looseness; (iv) peculiar word; (v) peculiar sentence; (vi) peculiar logic; (vii) perservation; and (viii) distractibility [58]. These items were summarized into the domains Impoverished and Disorganization, according to Liddle et al. [58].

### Feature extraction using natural language processing

Linguistic variables were extracted from transcripts using Python (version 3.11) and spaCy, which is an advanced library particularly well suited for the German language (https://github.com/explosion/spaCy; https://spacy.io/models). We employed the German transformer pipeline (de_dep_news_trf-3.7.2), a transformer-based model that can more effectively capture and process contextual information (https://spacy.io/models). This model was utilized to extract predefined linguistic features across various linguistic levels [59]. See Supplementary Material 1 and Supplementary eTable 1 for details on the extracted features.

### Statistical analyses

All analyses were conducted in R (version 4.3.1). To investigate group differences between MDD, BD, SSD and HC, we used one-way analysis of variance (ANOVA), followed by a post-hoc analysis with Bonferroni correction for multiple testing. The Kruskal–Wallis test was employed when the assumption for parametric testing was not satisfied [60] (cf. Table 1). The same procedure was conducted for the extracted linguistic features (cf. Table 2).

**Table 2.** Group comparison of extracted linguistic variables

	Major depressive disorder(*n* = 119)	Bipolar disorder(*n* = 27)	Schizophrenia spectrum disorders(*n* = 48)	Healthy controls(*n* = 178)	Group comparison	Effect size
Linguistic variables						
Adjective ratio	.02 (.01)	.02 (.01)	.02 (.00)	.02 (.01)	*p* = .043 (F = 2.73)	*η^2^* = .021
Adposition ratio	.07 (.01)	.07 (.01)	.07 (.01)	.07 (.01)	*p* = .060 (F = 2.48)	*η^2^* = .019
Adverb ratio	.18 (.03)	.17 (.03)	.18 (.03)	.18 (.03)	*p* = .512 (F = .76)	*η^2^* = .006
Auxiliary ratio	.06 (.01)	.07 (.01)	.06 (.01)	.06 (.01)	*p* = .689 (F = .48)	*η^2^* = .004
Coordinating conjunction ratio	.05 (.01)	.05 (.01)	.05 (.01)	.05 (.01)	*p* = .840 (F = .27)	*η^2^* = .002
Determiner ratio	.10 (.00)	.11 (.07)	.10 (.02)	.11 (.02)	*p* = .249 (F = 1.37)	*η^2^* = .011
Interjection ratio	.01 (.01)	.01 (.00)	.01 (.01)	.01 (.01)	*p* = .199 (H = 4.64)	*η^2^* = .006
Noun ratio	.13 (.02)	.14 (.03)	.13 (.02)	.14 (.02)	*p* = .386 (F = 1.01)	*η^2^* = .008
Particle ratio	.03 (.01)	.03 (.01)	.03 (.01)	.03 (.01)	*p* = .942 (F = .12)	*η^2^* = .001
Pronoun ratio	.13 (.02)	.14 (.03)	.14 (.03)	.13 (.02)	*p* = .053 (F = 2.57)	*η^2^* = .020
Proper noun ratio	.01 (.01)	.01 (.00)	.01 (.01)	.01 (.01)	*p* = .714 (H = 1.36)	*η^2^* = .007
Subordinating conjunction ratio	.02 (.01)	.02 (.00)	.02 (.01)	.02 (.00)	*p* = .907 (F = .18)	*η^2^* = .001
Verb ratio	.12 (.01)	.12 (.01)	.12 (.01)	.12 (.01)	*p* = .080 (F = 2.26)	*η^2^* = .018
Filled pause ratio	.02 (.02)	.02 (.01)	.02 (.01)	.02 (.02)	*p* = .147 (H = 5.36)	*η^2^* = .014
Noun-verb ratio	1.13 (.27)	1.12 (.27)	1.12 (.32)	1.19 (.30)	*p* = .169 (F = 1.68)	*η^2^* = .013
Open-closed ratio	.97 (.09)	.94 (.06)	.97 (.11)	.98 (.11)	*p* = .389 (F = 1.00)	*η^2^* = .008
Type-token ratio	.32 (.05)	.34 (.06)	.32 (.04)	.31 (.04)	*p* = .128 (F = 1.90)	*η^2^* = .015
Root overlap	.29 (.17)	.26 (.18)	.24 (.14)	.31 (20)	*p* = .098 (H = 6.29)	*η^2^* = .016
Mean length of utterance	16.84 (9.97)	18.05 (11.99)	13.85 (4.95)	18.48 (12.46)	*p* = .013 (H = 10.64)	*η^2^* = .019
Simple sentence ratio	.70 (.69)	.76 (.21)	.75 (.24)	.72 (.52)	*p* < .001 (H = 18.50)	*η^2^* = .001
Syntactic complexity	.32 (.15)	.37 (.18)	.28 (.09)	.34 (.21)	*p* = .146 (F = 1.80)	*η^2^* = .014
Syntactic diversity	.27 (.12)	.30 (.08)	.26 (.10)	.28 (.14)	*p* = .570 (F = .67)	*η^2^* = .005
Mean dependency distance	3.36 (.04)	3.31 (.46)	3.09 (.40)	3.38 (.48)	***p* = .002**ᵃ (F = 5.01)	*η^2^* = .039
Similarity mean (FastText)	.33 (.01)	.33 (.02)	.33 (02)	.33 (.01)	*p* = .835 (F = .28)	*η^2^* = .002
Coherence mean (BERT)	.54 (.05)	.54 (.05)	.53 (.05)	.54 (.04)	*p* = .848 (F = .26)	*η^2^* = .002
Coherence mean (BioLord)	.30 (.03)	.30 (.02)	.30 (.02)	.29 (.02)	*p* = .221 (F = 1.47)	*η^2^* = .011
Hohenheim index	10.84 (1.72)	10.62 (2.14)	9.87 (1.54)	11.18 (1.84)	***p* < .001**ᵃ (F = 6.91)	*η^2^* = .053
Probability negative	.84 (.10)	.85 (.08)	.82 (.09)	.84 (.09)	*p* = .479 (H = 2.47)	*η^2^* = .005

*Note:* Means (standard deviation), *F* = F-statistic from one-way ANOVAs; *H* = H-statistic from Kruskal–Wallis tests (used when ANOVA assumptions were not met); *η^2^* = Eta-squared, an effect size measure for ANOVA, indicating the proportion of variance in the dependent variable explained by group differences. Post hoc correction for multiple testing (Bonferroni) with significant results **in bold font**.

ᵃ = HC > SSD.

Next, we employed network analyses using R to investigate the structural and relational properties, aiming to elucidate patterns of interplay across linguistics, psychopathology, and cognition. Analyses included 28 linguistic variables (cf. Supplementary eTable 1), 10 psychopathological variables, and 6 cognitive variables (cf. Table 1). All variables were z-standardized. Networks were estimated using the Extended Bayesian Information Criterion Graphical Least Absolute Shrinkage and Selection Operator (*EBICglasso*) method [61, 62], with a tuning parameter (γ) set at 0.5. This tuning parameter controls the balance between sparsity and sensitivity in edge selection, emphasizing the most robust associations, while reducing the inclusion of spurious edges, based on prior recommendations for psychological and behavioral data [63, 64]. To assess the stability and robustness of the network structure, we conducted nonparametric bootstrapping with 1,000 permutations, generating confidence intervals for edge weights and evaluating network stability across resampled data. Insignificant edges were pruned to enhance network sparsity, retaining only stable and meaningful connections. Edges were classified as domain-specific (within linguistic, cognitive, or psychopathological domains) or cross-domain. Separate networks were estimated for (i) total sample (transdiagnostic), (ii) patients only, (iii) healthy controls only, (iv) each diagnostic group individually, and (v) the total sample excluding psychopathological variables as a supplementary analysis (Supplementary Material 3, Supplementary eTable 7–8).

To compare network structures, we employed the Network Comparison Test (NCT) from the *NCT R package (version 2.2.2.).* The NCT investigates network invariance by evaluating differences in overall network structures as well as global strength [65]. Test statistics and corresponding *p*-values were computed to determine whether the compared networks differed significantly. In addition to comparing healthy controls with the pooled patient groups, we conducted three pairwise NCTs to assess potential differences across diagnostic subgroups: MDD vs. BD, MDD vs. SSD, and BD vs. SSD. To account for multiple testing across edges, Bonferroni correction was applied to all *p*-values from the edge-specific comparisons. Additionally, Frobenius distances between group-level networks were calculated to quantify overall structural similarity [66].

## Results

### Sample characteristics

Demographic data are presented in Table 1. Group comparisons of the linguistic variables (Table 2) revealed several significant differences. SSD patients produced the simplest syntactic structures, BD patients showed the highest simple sentence ratio, and HC the most complex sentences. Adjective use differed significantly between groups, while pronoun use showed a trend. No significant group differences were found for the remaining linguistic variables (see Table 2).

### Network analyses

**Figure 1.**
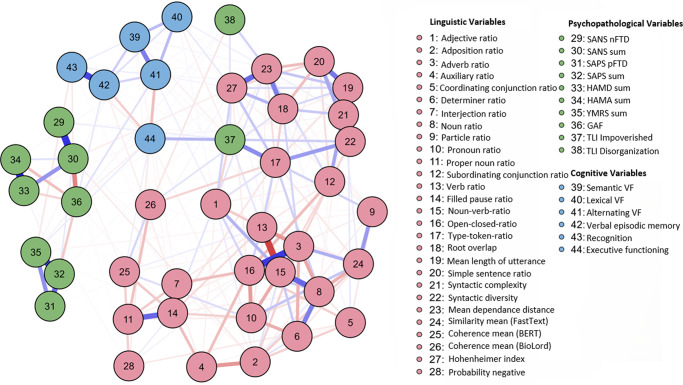
Network plot over all participants (total sample). *Note*: Node color represents domain (green = psychopathological variables, blue = cognitive variables, red = linguistic variables). Edge color represents correlation (blue = positive; red = negative association). Edge thickness indicates strength of association. The maximum strength of the edges was .63.

Network analyses were used to examine interaction patterns across linguistic, psychopathological, and cognitive domains. The full sample network included 44 nodes (28 linguistic, 10 psychopathological, and 6 cognitive) and was estimated via EBICglasso (γ = .5). In total, 282 of the 946 possible edges were non-zero (sparsity = .7019), indicating a relatively dense network (Figure 1). Maximum edge weight (EW) was .63.

#### Network clustering

The linguistic domain (red nodes) formed the most cohesive cluster, with high interconnections between variables reflecting lexical diversity, syntactic complexity, semantic coherence, and speech disfluencies. Notably, noun–verb ratio (NVR, no. 15) appeared as a central hub within this cluster. The psychopathological domain (green nodes) comprised a cohesive subgroup with limited cross-domain ties. Both TLI variables – TLI Impoverished (no. 37) and TLI Disorganization (no. 38) – diverged from this pattern: TLI Impoverished bridged the domains, while TLI Disorganization remained largely isolated. The cognition domain (blue nodes) showed strong domain-specific connections, especially among both VLMT variables (verbal episodic memory, no. 42; recognition, no. 43).

#### Edge strength and key connections

The strongest positive edge was observed between filled pause ratio (no. 14) and interjection ratio (no. 7; EW = .63), which is expectable for a verbalized thinking process. Additional prominent positive connections included verbal episodic memory and recognition (no. 42 and no. 43; EW = .56), HAM-D and HAM-A (no. 33 and no. 34; EW = .56), and adverb ratio with open–closed ratio (no. 3 and no. 16; EW = .52). The strongest negative edge emerged between verb ratio (no. 13) and NVR (no. 15; EW = -.50), underscoring their inverse relationship. Bridging variables across domains included executive functioning (no. 44), coherence mean (BioLord, no. 26), and TLI Impoverished (no. 37).

#### Centrality measures

The analysis of centrality measures revealed the pivotal roles of various variables. TLI Impoverished and type–token ratio (TTR) exhibited the highest betweenness, indicating strong integrative roles. Mean length of utterance (MLU), open-closed ratio, adverb ratio, and syntactic complexity showed the highest strength and expected influence, underlining their centrality in the network. SAPS (sum) and SANS (sum) revealed moderate expected influence, whereas HAM-D, HAM-A, and GAF were peripheral. Negative expected influence values were observed for several linguistic features (e.g., pronoun and auxiliary ratios), suggesting potential inhibitory associations. See Table 3 for detailed results.

**Table 3.** Centrality measures per variable (total sample)

Variable	Network			
	Betweenness	Closeness	Strength	Expected influence
Adjective ratio	−0.7426	0.8647	−0.3844	−0.1969
Adposition ratio	−0.3900	0.2223	0.0283	−1.6797
Adverb ratio	1.0913	1.2299	2.0642	−0.9609
Auxiliary ratio	0.1743	0.4412	−0.8071	−2.0136
Coordinating conjunction ratio	−0.8132	−0.3886	−1.1389	−1.3217
Determiner ratio	−0.6192	0.4852	0.0678	−0.2580
Interjection ratio	−0.2665	0.4094	0.1910	−0.3510
Noun ratio	−0.2842	1.0124	1.5268	0.1597
Particle ratio	−0.5487	0.2420	−0.9215	−0.5660
Pronoun ratio	−0.7250	0.4575	0.2149	−2.1248
Proper noun ratio	−0.6897	0.0896	−0.6008	−0.6864
Subordinating conjunction ratio	−0.4429	0.5269	−0.4964	−0.8791
Verb ratio	0.2801	0.9999	0.8557	−1.8234
Filled pause ratio	0.4918	0.5233	1.7700	0.2543
Noun–verb ratio	−0.4252	0.8079	1.1720	−0.5699
Open–closed ratio	1.0208	1.0267	2.1063	−0.5720
Type–token ratio	3.7365	1.8622	1.2087	0.3601
Root overlap	−0.4605	0.9427	0.6535	0.4978
Mean length of utterance	−0.0373	0.7568	1.7606	2.0697
Simple sentence ratio	−0.8308	0.4379	0.5475	−0.3725
Syntactic complexity	−0.4252	0.5145	0.3874	1.5480
Syntactic diversity	0.3507	1.1745	0.0323	0.9769
Mean dependency distance	0.0862	0.9128	1.3493	0.9793
Similarity mean (FastText)	−0.4605	0.5561	0.2685	−0.3246
Coherence mean (BERT)	−0.5310	−0.6761	−1.0140	−0.7682
Coherence mean (BioLord)	−0.3547	0.1244	−1.3593	−0.7709
Hohenheim index	0.8973	1.0051	0.7227	0.8550
Probability negative	−0.7779	−1.2682	−1.7324	−0.8513
SANS nFTD	0.7739	−0.6097	−1.0501	0.6280
SANS sum	0.9150	−0.7185	0.3673	0.9642
SAPS pFTD	−0.8661	−1.6771	−0.6364	0.6113
SAPS sum	−0.5487	−1.4885	−0.0171	1.1313
HAMD sum	−0.2312	−1.1108	−0.2883	1.0870
HAMA sum	−0.7603	−1.2955	−0.6122	0.4622
YMRS sum	0.3507	−1.3243	−0.3029	0.7523
GAFscore	−0.3547	−1.4971	−0.8717	−1.0021
TLI Impoverished	3.6659	1.4137	0.1608	0.5609
TLI Disorganization	−0.8661	−0.3341	−1.7593	−0.2562
Semantic VF	−0.7250	−1.3417	−0.5441	0.9022
Lexical VF	0.2449	−1.1636	−1.0080	0.6039
Alternating VF	−0.0725	−1.2906	−0.1915	1.2760
Verbal episodic memory (VLMT)	−0.1078	−1.4511	0.0603	1.2242
Recognition (VLMT)	−0.7074	−1.5791	−0.4696	0.7205
Executive functioning (TMT_Diff)	0.9855	0.1750	−1.3096	−0.2759

*Note: Betweenness* = measures the extent to which a node lies on the shortest paths between others; *closeness* = reflects how quickly information spreads from a given node to others; *strength* = indicates the overall level of connectivity/influence a node has in the network; *expected influence* = combines edge weights and network structure to estimate a node’s potential impact on the network.

#### Network comparison

**Figure 2.**
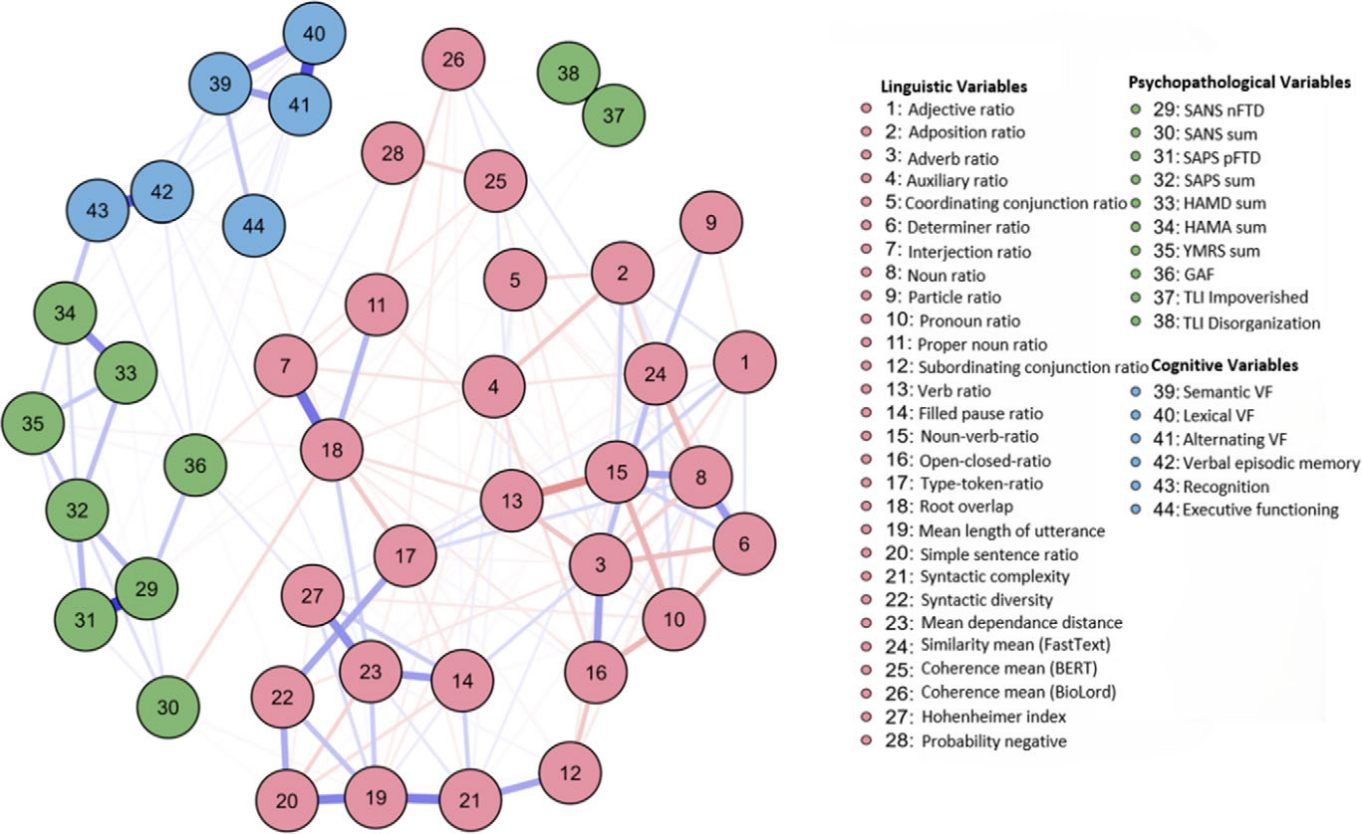
Network plot over all patients. *Note*: Node color represents domain (green = psychopathological variables, blue = cognitive variables, red = linguistic variables). Edge color represents correlation (blue = positive; red = negative association). Edge thickness indicates strength of association. The maximum strength of the edges was .51.

**Figure 3.**
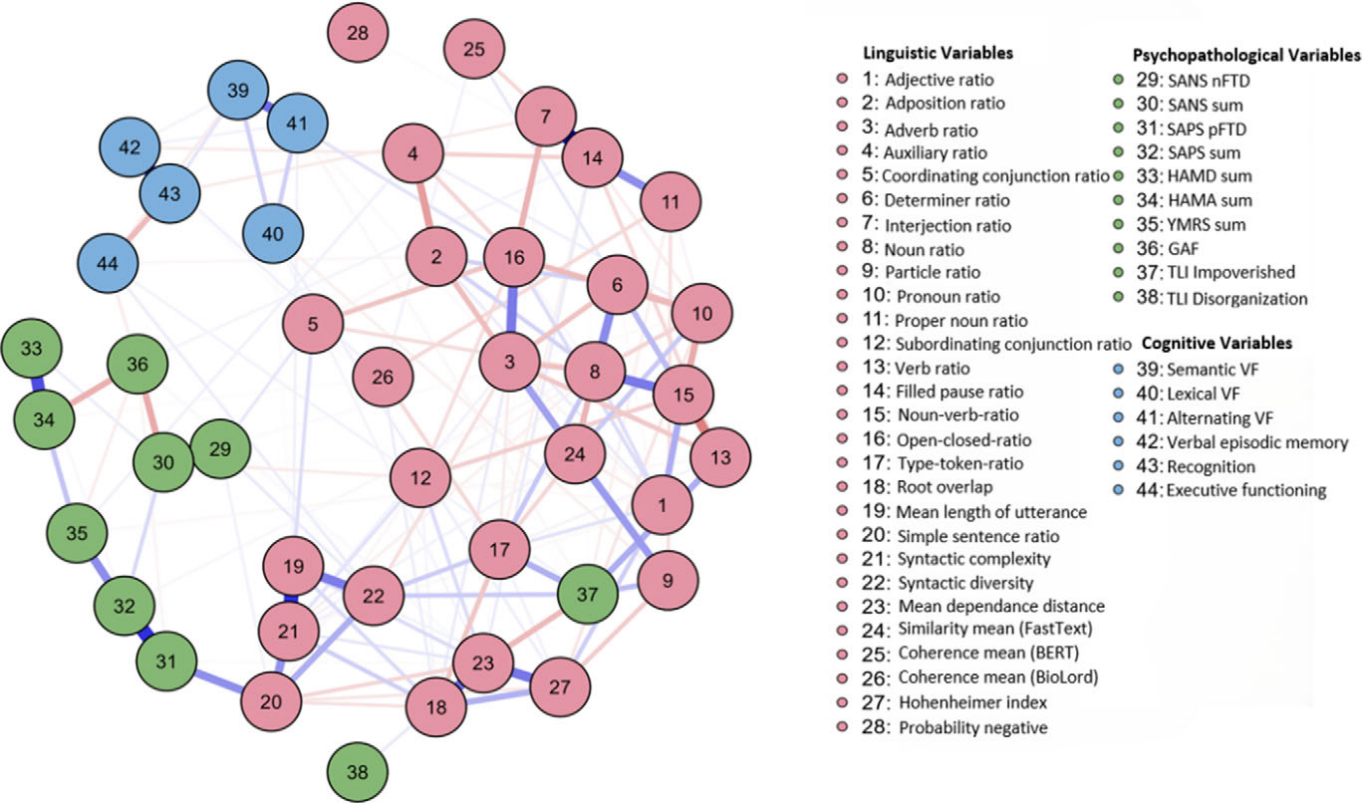
Network plot over all healthy controls. *Note*: Node color represents domain (green = psychopathological variables, blue = cognitive variables, red = linguistic variables). Edge color represents correlation (blue = positive; red = negative association). Edge thickness indicates strength of association. The maximum strength of the edges was .62.

Figure 2 (network plot over all patients) and Figure 3 (network plot over all healthy controls) show network properties across healthy controls and patients separately. By employing the NCT from the *NCT R package (version 2.2.2.)*, the networks’ structures of HC and patients were statistically compared. No significant differences were found in structure (*M* = 0.307, *p =* .335) or global strength (S = 1.329, *p =* .599), indicating structural invariance.

Further NCTs comparing diagnostic subgroups revealed a significant structural difference between MDD and SSD networks (*M* = 0.63, *p* = .033) but not between MDD and BD (*M* = 0.63, *p* = .499) nor between BD and SSD (*M* = 0.00, *p* = 1.00). After Bonferroni correction, no difference remained significant. Global strength did not differ significantly across any pairwise comparisons (all *p* > .65). Complementary Frobenius distance analyses supported these results, showing generally low numerical dissimilarity between networks. Full results for the separate networks of HC and patients, measurements of the NCT analyses, and centrality measures presented separately for patients and HC are provided in the Supplementary Material (Supplementary Material 2, Supplementary eTables 2–5; Frobenius results: Supplementary eTable 6).

## Discussion

This study investigated the interplay between language, cognition, and psychopathology within a transdiagnostic sample of patients with affective (MDD, BD) and psychotic (SSD) disorders and HC, using network analysis. Findings revealed linguistic variables, particularly measures of diversity and complexity, such as noun-verb ratio, type-token ratio, and mean length of utterance, emerged as central nodes linking linguistic, cognitive, and psychopathological domains. Cognitive performance showed strong associations with language impairments, while coherence measures derived from NLP served as key mediators between linguistic and psychopathological variables. These results suggest that, despite significant group differences in symptom load, the fundamental relationships among language, cognition, and psychopathology remain stable across groups. This underscores the potential of language-based assessments for better understanding of psychopathology across disorders and its dimensionality.

Based on this study, several new insights emerge. First, group comparisons revealed subtle yet significant linguistic differences between diagnostic groups. Notably, SSD patients exhibited reduced syntactic complexity, dependency distance, and mean length of utterance, aligning with prior research [30, 67]. Conversely, BD and SSD groups demonstrated higher simple sentence ratios, possibly reflecting compensatory mechanisms or reduced cognitive load [68]. Given that other linguistic measures exhibited less pronounced group differences, the findings suggest shared cognitive mechanisms underlying these patterns. The observed linguistic disruptions align with existing evidence linking language impairments to cognitive deficits and psychopathology. For instance, reduced syntactic diversity and coherence in SSD reflect impaired executive function and semantic network disturbances [5, 31, 69]. Similarly, the association between linguistic markers and mood symptoms in MDD and BD supports prior research on self-referential and emotionally valenced language in these disorders [37, 40].

Second, the findings of the network comparison suggest that the overall structure and global connectivity among linguistic, cognitive, and psychopathological variables did not significantly differ between patients and HC, nor across diagnostic groups, indicating that fundamental cross-domain relationships remain stable despite significant group differences in symptom load. This aligns with a dimensional perspective, suggesting that the structure of relationships among cognitive, psychopathological, and linguistic variables remains stable irrespective of categorical disease boundaries [70]. However, the more fragmented psychopathological domain in patients may indicate that psychiatric symptoms operate as more loosely connected constructs, aligning with research suggesting that psychopathology emerges from disruptions in broader cognitive networks rather than isolated deficits [14]. The presence of key linguistic bridging variables indicates potential compensatory mechanisms or structural differences in language organization. These results suggest that while linguistic patterns remain consistent, subtle differences in network connectivity may still contribute to individual variability in psychopathological expression. The network invariance across groups supports the notion that linguistic features play a central, dimensional role independent of a diagnosis and symptom load. The strong connections between linguistic variables in both groups underscore the stability of linguistic network architecture even in the presence of mental illness, without evidence for diagnosis-specific structural changes. Complementing these findings, additional within-patient analyses showed that despite subtle structural differences involving the MDD network, no robust, diagnosis-specific edges differentiate MDD, BD, and SSD, nor were any significant differences found in global strength. This pattern provides further support for a transdiagnostic, dimensional perspective, suggesting that the core architecture of symptom networks is largely shared across these major disorders. Diagnostic distinctions may therefore lie more in nuanced, higher-order network configurations than in specific symptom-to-symptom pathways. These findings align with dimensional and transdiagnostic models that conceptualize cognitive and linguistic impairments as shared mechanisms across various psychopathologies [5, 70, 71].

Third, network analyses provided insights into how language, cognition, and psychopathology interplay across diagnoses. Linguistic variables formed the most densely connected cluster across all groups, with measures, such as noun-verb ratio, type-token ratio, and mean length of utterance, acting as key nodes. This reinforces the centrality of linguistic features in understanding psychiatric disorders. Additionally, bridging variables like semantic coherence, executive functioning, and TLI Impoverished linked linguistic, cognitive, and psychopathological domains, highlighting their transdiagnostic relevance, which aligns with dimensional models of psychopathology that emphasize overlapping features across traditional diagnostic boundaries [17, 70, 72]. Semantic coherence, often reduced in psychotic disorders, reflects underlying thought disorganization [1, 73]. Similarly, lexical complexity measures, such as mean length of utterance and type-token ratio, are central in characterizing language disruptions associated with affective and psychotic disorders [59]. Linguistic patterns have been linked to neurocognitive mechanisms and may serve as early indicators of cognitive decline or psychiatric progression [71]. Importantly, impairments in semantics and syntax often precede full-blown clinical symptoms in SZ and MDD [23, 25, 31, 74, 75]. Cognitive deficits, particularly in executive functioning, strongly interact with psychopathology [14, 76, 77]. Executive dysfunction, affecting planning, flexibility, and inhibition, is a key transdiagnostic feature across psychiatric disorders and emerged as a central link between cognitive, psychopathological, and linguistic variables [14]. Its association with TLI Impoverished suggests that language-related deficits may exacerbate broader cognitive impairments, aligning with dimensional models of psychopathology such as HiTOP [70]. Furthermore, cognitive impairments contribute to the persistence of negative symptoms in SZ and the recurrence of mood episodes in BD [76, 77]. The high centrality of linguistic variables highlights their relevance in psychiatric symptom networks. Decreased type-token ratio has been associated with cognitive and social dysfunction in SZ, while increased syntactic complexity may reflect compensatory mechanisms in affective disorders [31, 78]. These findings suggest that aspects of linguistic diversity and structure may serve as transdiagnostic markers of cognitive and functional impairments. Moreover, they could hold prognostic value concerning symptom severity and disease progression across psychiatric disorders, aligning with dimensional models of psychopathology [17].

In summary, the results indicate that linguistic networks remain stable across HC and psychiatric diagnoses, while psychopathological symptoms exhibit some structural variability. This underscores the importance of a dimensional and transdiagnostic approach that considers linguistic and cognitive mechanisms as common factors in mental disorders.

## Limitations

Despite the notable sample size, several limitations should be acknowledged. As this study employed a cross-sectional design, conclusions regarding the longitudinal stability or causal direction of the observed associations cannot be drawn. The patient sample covered a broad range of illness phases from acute to remitted states, but highly acute patients could not be included due to practical constraints of participation during severe acute states. Future studies should employ longitudinal designs capturing different illness phases and symptom fluctuations to examine whether the identified linguistic, cognitive, and psychopathological associations remain stable over time or vary with changes in clinical states. Such approaches would also allow testing the predictive validity and temporal robustness of the present network structures.

The diagnostic distribution was unbalanced, with fewer participants with bipolar and psychotic disorders than with depression, which may limit generalizability. However, NCTs revealed no significant group differences, suggesting that core linguistic–cognitive–psychopathological patterns are shared across diagnoses.

Finally, speech data were derived from a single task (TAT, [57]), which restricted variability but ensured standardization. Additionally, psychopathological scales may not be equally sensitive or meaningful in HCs as they are primarily designed to capture clinical symptomatology in patients. To address this potential limitation, an additional network excluding psychopathological variables showed no differences from the original network (Supplementary Material 3, eTable 7–8).

## Conclusion

This study highlights the intricate interplay of linguistic, cognitive, and psychopathological features, emphasizing their roles within a transdiagnostic framework. Results underscore the importance of integrating linguistic assessments into psychiatric evaluations and call for further exploration of the causal mechanisms underlying these associations.

## Supporting information

10.1192/j.eurpsy.2026.10151.sm001 Mülfarth et al. supplementary material Mülfarth et al. supplementary material

## Data Availability

The data supporting the findings of this study can be accessed by contacting the corresponding author. All codes used for linguistic feature extraction and network analyses are available at https://github.com/NeuroSTA/NeuroSTA.

## Supplementary material

The supplementary material for this article can be found at http://doi.org/10.1192/j.eurpsy.2026.10151.
