# Supplementary material for: Linking spontaneous speech, cognition, and psychopathology across affective and psychotic disorders: A network approach
Source: Eur Psychiatry. 2026 Jan 23;69(1):e21. doi: 10.1192/j.eurpsy.2026.10151 (PMC12925669; doi:10.1192/j.eurpsy.2026.10151)
Supplement: Mülfarth et al. supplementary material [file S0924933826101515sup001.docx]

**Supplementary Material:**

*1. Feature Extraction Using Natural Language Processing*

To measure linguistic diversity, we extracted the ratios of different word classes: (i) adjectives, (ii) adpositions, (iii) adverbs, (iv) auxiliaries, (v) coordinating conjunctions, (vi) determiners, (vii) interjections, (viii) nouns, (ix) particles, (x) pronouns, (xi) proper nouns, (xii) subordinating conjunctions, and (xiii) verbs. These part-of-speech (POS) ratios were calculated by dividing the frequency of each POS by the total number of words used. Also, the ratio of filled pauses was conducted in the same way, which indicate speech disfluencies (e.g., ‘hm’). Further features used to measure lexical diversity were type-token-ratio (TTR), noun-verb-ratio (NVR), and open-closed-ratio (OCR). TTR was measured by dividing the number of unique words (types) by the total number of words (tokens) and can give an indication of the variety and richness of the speaker’s vocabulary. NVR calculates the ratio of verbs to nouns and in OCR, the use of open word classes (content words, e.g., adjectives) was measured compared to the use of closed word classes (function words, e.g., prepositions). These two can give an insight of how complex the speaker’s language is (De Boer et al., 2020).

At the syntactic level, the mean length of an utterance (MLU), the ratio of simple sentences, the syntactic complexity and the syntactic diversity were extracted. MLU is calculated as the average number of words per utterance (in this case, per sentence) by dividing the total number of words by the total number of sentences. The simple sentence ratio represents the proportion of main clauses without embedded sentences, determined by dividing the number of simple sentences by the total number of sentences. Syntactic complexity was assessed by the ratio of complex sentences, calculated as the number of main clauses containing subordinate clauses divided by the total number of sentences. Syntactic diversity was measured as the ratio of different types of complex sentences, determined by dividing the number of distinct complex sentence types by the total number of complex sentences [33]. Using spaCy’s dependency parser, mean dependency distance was assessed by quantifying syntactic complexity, which combines average sentence length and the depth of syntactic dependency trees (distance between heads and their dependents). Using spaCy’s lemmatizer, the average proportion of shared lemmas (morphological roots) between all pairs of sentences was calculated to determine morphological root overlap. Also, a readability score called Hohenheim index was calculated, which assesses text difficulty and cognitive load by considering factors such as sentence length and syllables per word [81].

In addition to the use of spaCy’s transformer-based model, word embeddings from the German FastText model were used to extract the similarity mean by computing the cosine similarity between consecutive sentences by capturing word-level semantics [82]. Moreover, the coherence mean was extracted in two ways. For one, Bidirectional Encoder Representations from Transformers (BERT) embeddings from a pre-trained German BERT model were used (deepset, 2019). The latter captures profound contextual relationships between sentences by considering the full context allowing for the evaluation of nuanced coherence. Using BERT, coherence mean was extracted. Coherence mean was also extracted using BioLord. BioLord-2023 is a multilingual model with official fine-tuning in – among others – German, tailored for the biomedical and clinical context by combining large language models (LLMs) with insights from clinical knowledge graphs [83]. By using XLM-RoBERTa-German (https://huggingface.co/ssary/XLM-RoBERTa-German-sentiment), sentiment analysis was conducted to estimate probability of negative sentiment in speech (probability negative). These linguistic features were included because of their capability to reveal subtle language impairments associated with psychiatric disorders and thus their importance for psychiatric research [33,76,84]. See following eTable 1 for an overview of all extracted features and their definitions.

2. *Comparison of Networks in Healthy Controls and Patients Only*

Figure 2 (network plot over all patients) and Figure 3 (network plot over all healthy controls) show network properties across healthy controls and patients separately. In the network plot over all patients (Figure 2), the linguistic domain (red nodes) also formed the densest cluster with strong interconnections. 204 of 946 possible edges were non-zero (sparsity = .7844). The maximum strength of the edges was .51 (positive correlation between HAM-D sum, no. 33, and HAM-A sum, no. 34). Linguistic variables such as noun-verb-ratio (no. 15), verb ratio (no. 13), and open-closed-ratio (no.16) formed a tightly interconnected subgroup, highlighting lexical characteristics as a core component. The psychopathological domain (green nodes) appeared more fragmented, forming a cohesive subgroup of eight variables with strong internal connections, but limited cross-domain links. A similar pattern was observed for the cognitive domain. In the network over all healthy controls (Figure 3), the linguistic domain (red nodes) again formed a well-connected cluster. 165 of 946 possible edges were non-zero (sparsity = .8256). The maximum strength of the edges was .62 (positive correlation between verbal episodic memory, no. 42, recognition, no. 43; and .61 between filled pause ratio, no. 14, and interjection ratio, no. 7). Bridging variables were coordinating conjunction ratio (no. 5) and simple sentence ratio (no. 20). The latter showed a strong connection to SAPS pFTD (no. 31) connecting the linguistic and the psychopathology domain. Notably, the psychopathological variable TLI Impoverished (no. 37) reappeared as a central node showing increased integration into the linguistic domain.

**eTable 1.** Extracted linguistic variables using NLP.

| Variable | Description |
| --- | --- |
| Adjective ratio | Proportion of adjectives used relative to total tokens (total number of words) |
| Adposition ratio | Proportion of adpositions used relative to total tokens |
| Adverb ratio | Proportion of adverbs used relative to total tokens |
| Auxiliary ratio | Proportion of auxiliaries used relative to total tokens |
| Coordinating conjunction ratio | Proportion of coordinating conjunctions used relative to total tokens |
| Determiner ratio | Proportion of determiner used relative to total tokens |
| Interjection ratio | Proportion of interjections used relative to total tokens |
| Noun ratio | Proportion of nouns used relative to total tokens |
| Particle ratio | Proportion of particles used relative to total tokens |
| Pronoun ratio | Proportion of pronouns used relative to total tokens |
| Proper noun ratio | Proportion of proper nouns used relative to total tokens |
| Subordinating conjunction ratio | Proportion of subordinating conjunctions used relative to total tokens |
| Verb ratio | Proportion of verbs used relative to total tokens |
| Filled pause ratio | Disfluencies while talking (pauses filled with fillers, e.g., ‘hm’); Proportion of filled pauses used relative to total tokens |
| Noun-verb-ratio | Ratio of verbs used compared to the number of nouns |
| Open-closed-ratio | Ratio of open word classes (content words e.g., adjectives) used compared to the number of closed word classes (function words e.g., prepositions) |
| Type-token-ratio | Ratio of total number of words (token) used compared to the number of different (unique) words |
| Root overlap | Morphological root overlap |
| Mean length of utterance | Average number of words per utterance (here sentence) |
| Simple sentence ratio | Ratio of main clauses without embedded sentences |
| Syntactic complexity | Ratio of main clauses embedding subordinate clauses (incl. conjunctions) |
| Syntactic diversity | Ratio of different types of complex sentences |
| Mean dependency distance | Quantifies syntactic complexity |
| Similarity mean (FastText) | Average cosine similarity between consecutive sentences based on word vectors, measuring coherence |
| Coherence mean (BERT) | Coherence based on BERT embeddings, assessing the semantic relationship between consecutive sentences |
| Coherence mean (BioLord) | Evaluates semantic coherence in medical-related discourse |
| Hohenheim index | Evaluates sentence complexity, syllable count, and polysyllabic words |
| Probability negative | Estimated probability of negative sentiment in speech, measured by sentiment analysis |

**eTable 2.** Centrality Measures per Variable (Patients only).

| Network | | | | |
| --- | --- | --- | --- | --- |
| Variable | Betweenness | Closeness | Strength | Expected Influence |
| Adjective ratio | -0.9364 | 0.1988 | -0.4011 | -0.6389 |
| Adposition ratio | 1.8065 | 0.8533 | 0.0228 | -1.1332 |
| Adverb ratio | 1.0918 | 1.3266 | 1.8092 | -1.1368 |
| Auxiliary ratio | -0.0285 | 0.6444 | -0.7617 | -1.8515 |
| Coordinating conjunction ratio | -0.9557 | -0.5633 | -1.5728 | -0.9778 |
| Determiner ratio | -0.9557 | 0.5617 | 0.7818 | -0.3055 |
| Interjection ratio | -0.7819 | 0.5138 | -0.2707 | -0.1272 |
| Noun ratio | -0.2217 | 0.9329 | 1.6899 | -0.0160 |
| Particle ratio | -0.9557 | -0.2324 | -1.4883 | -0.3724 |
| Pronoun ratio | -0.8398 | 0.8814 | 0.6246 | -2.4028 |
| Proper noun ratio | -0.6273 | 0.2251 | -1.0405 | -0.3132 |
| Subordinating conjunction ratio | -0.4149 | 0.4292 | -0.5454 | -0.7286 |
| Verb ratio | 0.3192 | 1.1830 | 0.1962 | -1.9304 |
| Filled pause ratio | 1.5361 | 0.9531 | 1.8219 | 0.0752 |
| Noun-verb-ratio | 0.4737 | 1.1172 | 1.5190 | -0.4919 |
| Open-closed-ratio | 0.0487 | 0.7679 | 0.4322 | -1.0565 |
| Type-token-ratio | 1.1111 | 1.2045 | 0.9219 | 0.2486 |
| Root overlap | 0.2226 | 1.1245 | 0.6338 | 0.8684 |
| Mean length of utterance | 0.2612 | 0.7454 | 1.8238 | 2.1078 |
| Simple sentence ratio | -0.9364 | 0.5392 | 0.2999 | 0.2103 |
| Syntactic complexity | -0.2990 | 0.5406 | 0.5693 | 1.4509 |
| Syntactic diversity | -0.2024 | 0.6708 | -0.0005 | 0.8893 |
| Mean dependency distance | 2.5019 | 1.3758 | 1.6851 | 1.2615 |
| Similarity mean (FastText) | 0.1067 | 0.8210 | 0.5861 | -0.3092 |
| Coherence mean (BERT) | -0.5308 | -0.5925 | -1.0954 | -1.0745 |
| Coherence mean (BioLord) | -0.9364 | -0.8107 | -1.4539 | -0.7515 |
| Hohenheim index | 2.0576 | 1.1504 | 0.2189 | 0.7736 |
| Probability negative | -0.7819 | -0.9498 | -1.6711 | -1.0194 |
| SANS nFTD | -0.9557 | -0.9944 | -1.0883 | 0.4650 |
| SANS sum | 1.9997 | -0.6500 | 0.6972 | 0.7935 |
| SAPS pFTD | -0.9557 | -1.6784 | -0.4688 | 0.6480 |
| SAPS sum | 0.1646 | -1.4003 | 0.4170 | 1.0557 |
| HAMD sum | -0.1444 | -1.1192 | 0.0407 | 1.0887 |
| HAMA sum | -0.9557 | -1.3719 | -0.6014 | 0.6005 |
| YMRS sum | -0.6273 | -1.5262 | 0.0102 | 0.7366 |
| GAFscore | 2.0190 | -0.4138 | -0.4897 | -0.6614 |
| TLI Impoverished | 0.4930 | 0.8136 | -0.0657 | 0.3347 |
| TLI Disorganization | -0.9557 | -0.9936 | -1.9911 | -0.4213 |
| Semantic VF | -0.8978 | -1.3293 | -0.1699 | 1.1548 |
| Lexical VF | -0.9557 | -1.5840 | -0.7134 | 0.5888 |
| Alternating VF | 0.6862 | -1.0050 | 0.0364 | 1.1230 |
| Verbal episodic memory (VLMT) | -0.5308 | -1.4524 | 0.4039 | 1.3342 |
| Recognition (VLMT) | -0.3376 | -1.3736 | -0.1505 | 0.5577 |
| Executive functioning (TMT_Diff) | 0.8214 | 0.4666 | -1.2013 | -0.6470 |

Note: *Betweenness* = measures the extent to which a node lies on the shortest paths between others; *closeness* = reflects how quickly information spreads from a given node to others; *strength* = indicates the overall level of connectivity/influence a node has in the network; *expected influence* = combines edge weights and network structure to estimate a node’s potential impact on the network.

**eTable 3.** Centrality Measures per Variable (Healthy Controls only).

| Network | | | | |
| --- | --- | --- | --- | --- |
| Variable | Betweenness | Closeness | Strength | Expected Influence |
| Adjective ratio | -0.5882 | 0.5796 | -0.0820 | 0.1327 |
| Adposition ratio | -0.7006 | 0.2713 | 0.0961 | -1.5443 |
| Adverb ratio | 1.1721 | 1.1713 | 1.5509 | -0.9694 |
| Auxiliary ratio | -0.5133 | 0.1457 | -0.6702 | -1.6339 |
| Coordinating conjunction ratio | -0.3635 | 0.3407 | -1.0719 | -1.0049 |
| Determiner ratio | -0.9065 | 0.4157 | 0.5908 | -0.3617 |
| Interjection ratio | 0.9474 | 0.6424 | 0.0636 | 0.0227 |
| Noun ratio | 0.0672 | 0.9165 | 1.8260 | 0.0837 |
| Particle ratio | -0.1949 | 0.8530 | -0.5073 | -0.5298 |
| Pronoun ratio | -1.0002 | 0.3973 | 0.5488 | -2.1950 |
| Proper noun ratio | 0.0111 | 0.1471 | -0.7507 | -0.4951 |
| Subordinating conjunction ratio | -1.0002 | 0.3897 | -0.8177 | -1.0444 |
| Verb ratio | -0.6818 | 0.7333 | 0.0634 | -1.6647 |
| Filled pause ratio | 1.4718 | 0.6270 | 1.4527 | 0.4260 |
| Noun-verb-ratio | -0.2324 | 0.7709 | 1.3475 | -0.6576 |
| Open-closed-ratio | 1.4156 | 1.0082 | 0.9111 | -1.2594 |
| Type-token-ratio | 0.3107 | 1.0748 | 0.5514 | -0.4168 |
| Root overlap | -0.4758 | 0.9169 | 0.7850 | 0.7118 |
| Mean length of utterance | -0.3447 | 0.6318 | 1.0908 | 2.0030 |
| Simple sentence ratio | 2.2583 | 0.7892 | 1.1014 | 0.5232 |
| Syntactic complexity | -0.5320 | 0.5747 | 0.9529 | 1.6448 |
| Syntactic diversity | 1.9961 | 0.8977 | 0.4075 | 1.0560 |
| Mean dependency distance | 1.8837 | 1.2753 | 1.3678 | 1.0176 |
| Similarity mean (FastText) | -0.3447 | 0.8614 | 0.9458 | -0.5947 |
| Coherence mean (BERT) | -1.0189 | -0.9489 | -1.6039 | -0.7604 |
| Coherence mean (BioLord) | -0.9815 | -0.2822 | -1.3118 | -1.0037 |
| Hohenheim index | 2.2957 | 1.1643 | 0.6265 | 0.3949 |
| Probability negative | -1.0189 | -2.4867 | -2.0265 | -0.7214 |
| SANS nFTD | -0.5507 | -0.8012 | -0.2051 | 0.8894 |
| SANS sum | -0.7193 | -0.8370 | -0.1112 | 0.7791 |
| SAPS pFTD | 1.4530 | 0.3295 | 0.2383 | 1.2256 |
| SAPS sum | 0.9661 | 0.0911 | 0.2882 | 1.1655 |
| HAMD sum | -0.8878 | -0.9854 | -0.7039 | 0.4608 |
| HAMA sum | -0.0077 | -0.7837 | -0.4055 | 0.4676 |
| YMRS sum | 0.4043 | -0.2469 | -0.4823 | 0.5272 |
| GAFscore | -0.7755 | -1.8538 | -1.7272 | -0.5895 |
| TLI Impoverished | -0.6631 | 0.9156 | -0.4441 | 0.1312 |
| TLI Disorganization | -1.0189 | -1.5863 | -2.0027 | -0.6555 |
| Semantic VF | -1.0189 | -1.4086 | -0.1392 | 0.8918 |
| Lexical VF | 1.0410 | -1.0515 | -0.6433 | 0.4605 |
| Alternating VF | 0.0298 | -1.3007 | 0.1564 | 1.2000 |
| Verbal episodic memory (VLMT) | 0.1234 | -1.4310 | 0.4712 | 1.4706 |
| Recognition (VLMT) | -0.2886 | -1.4590 | 0.2171 | 1.0851 |
| Executive functioning (TMT_Diff) | -1.0189 | -1.4692 | -1.9448 | -0.6687 |

Note: *Betweenness* = measures the extent to which a node lies on the shortest paths between others; *closeness* = reflects how quickly information spreads from a given node to others; *strength* = indicates the overall level of connectivity/influence a node has in the network; *expected influence* = combines edge weights and network structure to estimate a node’s potential impact on the network.

**eTable 4.** Results of Network Comparison Test (NCT) of Healthy Controls and Patients.

| **NETWORK INVARIANCE TEST**  Test statistic M: 0.3079656  *p*-value 0.3356643 | | |
| --- | --- | --- |
| **GLOBAL STRENGTH INVARIANCE TEST**  Global strength per group: 18.30166 (HC), 19.63159 (patients)  Test statistic S: 1.329931 | | |
| *p*-value 0.5994006 | | |
|  | | |
| **CENTRALITY INVARIANCE TEST** | ***p*-value** | |
|  | Strength | Expected influence |
| Adjective ratio | 0.23976024 | 0.18281718 |
| Adposition ratio | 0.46353646 | 0.25874126 |
| Adverb ratio | 0.38961039 | 0.66433566 |
| Auxiliary ratio | 0.83516484 | 0.96003996 |
| Coordinating conjunction ratio | 0.57442557 | 0.58741259 |
| Determiner ratio | 0.38461538 | 0.80519481 |
| Noun ratio | 0.17182817 | 0.90409590 |
| Particle ratio | 0.09390609 | 0.43956044 |
| Pronoun ratio | 0.68431568 | 0.65334665 |
| Subordinating conjunction ratio | 0.97302697 | 0.56143856 |
| Verb ratio | 0.84015984 | 0.69030969 |
| Filled pause ratio | 0.70029970 | 0.57642358 |
| Noun-verb-ratio | 0.88511489 | 0.75524476 |
| Open-closed-ratio | 0.21778222 | 0.55944056 |
| Type-token-ratio | 0.41158841 | 0.90409590 |
| Root overlap | 0.31668332 | 0.78421578 |
| Mean length of utterance | 0.54045954 | 0.99500500 |
| Simple sentence ratio | 0.26073926 | 0.34165834 |
| Syntactic complexity | 0.09490509 | 0.16883117 |
| Syntactic diversity | 0.53946054 | 0.74625375 |
| Mean dependency distance | 0.94205794 | 0.59840160 |
| Similarity mean (FastText) | 0.25174825 | 0.70629371 |
| Coherence mean (BERT) | 0.65634366 | 0.30869131 |
| Coherence mean (BioLord) | 0.38461538 | 0.56043956 |
| Hohenheim index | 0.11488511 | 0.96803197 |
| Probability negative | 0.98201798 | 0.77622378 |
| SANS nFTD | 0.05994006 | 0.07492507 |
| SANS sum | 0.75924076 | 0.62037962 |
| SAPS pFTD | 0.64935065 | 0.41058941 |
| SAPS sum | 0.16083916 | 0.01898102 |
| HAMD sum | 0.48051948 | 0.48251748 |
| HAMA sum | 0.67032967 | 0.83716284 |
| YMRS sum | 0.89110889 | 0.75824176 |
| GAFscore | 0.36663337 | 0.36563437 |
| TLI Impoverished | 0.61938062 | 0.63836164 |
| TLI Disorganization | 0.26973027 | 0.75024975 |
| Semantic VF | 0.42257742 | 0.39560440 |
| Lexical VF | 0.61038961 | 0.61838162 |
| Alternating VF | 0.50249750 | 0.50449550 |
| Verbal episodic memory (VLMT) | 0.90309690 | 0.79820180 |
| Recognition (VLMT) | 0.13386613 | 0.18781219 |
| Executive functioning (TMT_Diff) | 0.93006993 | 0.37262737 |

**BOLD** significant after correction for multiple testing.

**eTable 5.** Results of Network Comparison Test (NCT) across Diagnoses.

MDD and BD

| **NETWORK INVARIANCE TEST**  Test statistic M: 0.6294364  *p*-value 0.4985015 |
| --- |
| **GLOBAL STRENGTH INVARIANCE TEST**  Global strength per group: 11.20467 (MDD), 0 (BD)  Test statistic S: 11.20467 |
| *p*-value 0.6523477 |

MDD and SSD

| **NETWORK INVARIANCE TEST**  Test statistic M: 0.6294364  *p*-value 0.03296703 |
| --- |
| **GLOBAL STRENGTH INVARIANCE TEST**  Global strength per group: 11.20467 (MDD), 0 (SSD)  Test statistic S: 11.20467 |
| *p*-value 0.7092907 |

BD and SSD

| **NETWORK INVARIANCE TEST**  Test statistic M: 0  *p*-value 1 |
| --- |
| **GLOBAL STRENGTH INVARIANCE TEST**  Global strength per group: 0 (BD), 0 (SSD)  Test statistic S: 0 |
| *p*-value 1 |

**BOLD** significant after correction for multiple testing.

**eTable 6.** Frobenius Distances between Group Networks.

| **Group 1** | **Group 2** | **Frobenius** |
| --- | --- | --- |
| HC | Patients | 5.198810 |
| HC | MDD | 5.337742 |
| MDD | BD | 9.746464 |
| HC | BD | 9.784028 |
| HC | SSD | 11.527816 |
| MDD | SSD | 11.809872 |
| BD | SSD | 14.098795 |

Note: sorted by ascending Frobenius value. Lower Frobenius distances indicate higher numerical similarity between network structures.

*3. Network plot over all participants (total sample) excluding psychopathological variables.*


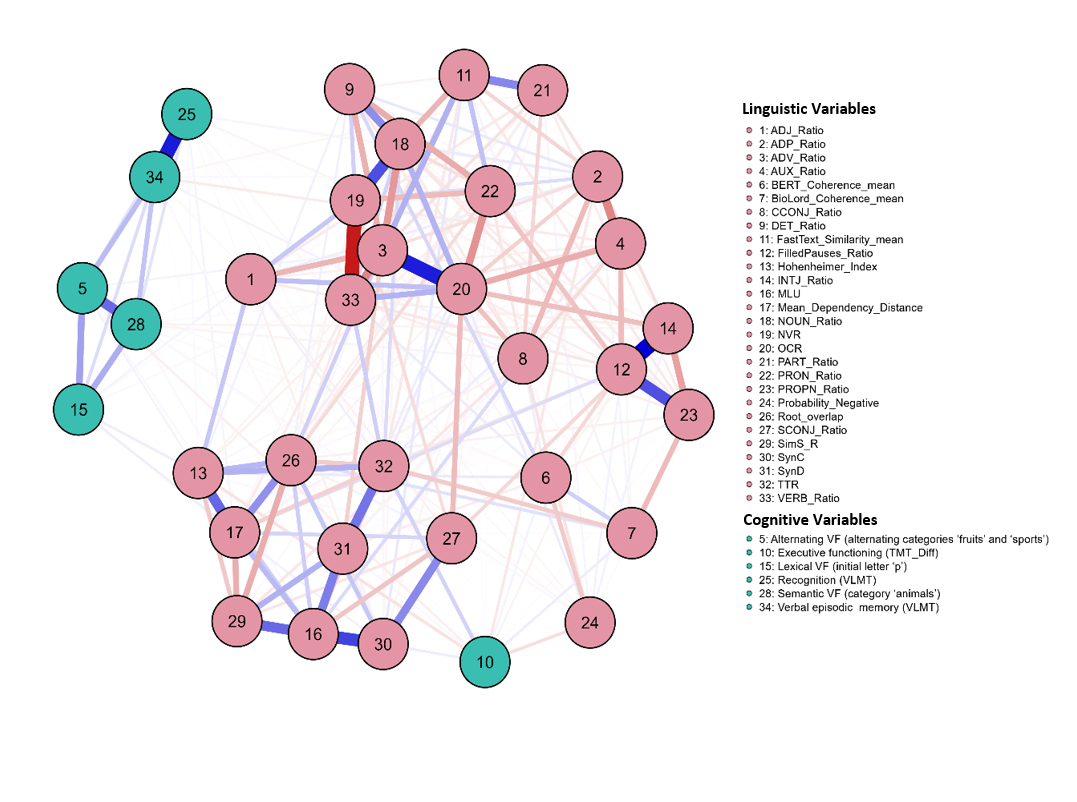


Note: Node color represents domain (green = cognitive variables, red = linguistic variables). Edge color represents correlation (blue = positive; red = negative association). Edge thickness indicates strength of association.

**eTable 7.** Centrality Measure per Variable (Total Sample **without** Psychopathology).

|  | | Network | | | | | | | |
| --- | --- | --- | --- | --- | --- | --- | --- | --- | --- |
| Variable | | Betweenness | | Closeness | | Strength | | Expected influence | |
| Adjective ratio |  | -0.425 |  | 0.430 |  | -0.764 |  | 0.026 |  |
| Adposition ratio |  | -0.647 |  | 0.251 |  | 0.005 |  | -1.403 |  |
| Adverb ratio |  | 2.681 |  | 1.610 |  | 1.986 |  | -0.805 |  |
| Auxiliary ratio |  | 2.143 |  | 0.920 |  | -0.447 |  | -1.849 |  |
| Alternating VF |  | -0.235 |  | -1.948 |  | -0.654 |  | 1.189 |  |
| Coherence mean (BERT) |  | -0.457 |  | -0.714 |  | -1.084 |  | -0.273 |  |
| Coherence mean (BioLord) |  | -0.425 |  | -0.260 |  | -1.383 |  | -0.381 |  |
| Coordinating conjunction ratio |  | -0.679 |  | 0.158 |  | -0.451 |  | -1.398 |  |
| Determiner ratio |  | -0.425 |  | 0.357 |  | -0.277 |  | -0.128 |  |
| Executive functioning (TMT_Diff) |  | -0.615 |  | -0.758 |  | -1.592 |  | -0.337 |  |
| Similarity mean (FastText) |  | 0.050 |  | 0.169 |  | -0.134 |  | 0.121 |  |
| Filled pause ratio |  | 1.065 |  | 0.669 |  | 1.297 |  | 0.546 |  |
| Hohenheim index |  | 0.209 |  | 0.401 |  | 0.428 |  | 1.281 |  |
| Interjection ratio |  | -0.203 |  | 0.549 |  | 0.251 |  | -0.386 |  |
| Lexical VF |  | -0.013 |  | -1.964 |  | -1.351 |  | 0.801 |  |
| Mean length of utterance |  | 0.019 |  | 0.317 |  | 1.513 |  | 2.082 |  |
| Mean_dependency_distance |  | -0.045 |  | 0.386 |  | 0.689 |  | 0.769 |  |
| Noun ratio |  | -0.425 |  | 0.704 |  | 1.327 |  | 0.426 |  |
| Noun-verb-ratio |  | -0.647 |  | 0.573 |  | 0.787 |  | -0.394 |  |
| Open-closed-ratio |  | 3.220 |  | 1.645 |  | 2.379 |  | -0.123 |  |
| Particle ratio |  | -0.869 |  | -0.522 |  | -0.836 |  | -0.486 |  |
| Pronoun ratio |  | -0.584 |  | 0.844 |  | 0.066 |  | -1.912 |  |
| Proper noun ratio |  | -0.393 |  | 0.150 |  | -0.111 |  | -0.871 |  |
| Probability negative |  | -0.837 |  | -1.382 |  | -1.693 |  | -0.620 |  |
| Recognition (VLMT) |  | -0.901 |  | -1.936 |  | -0.916 |  | 0.894 |  |
| Root overlap |  | -0.393 |  | 0.467 |  | 0.483 |  | 0.485 |  |
| Subordinating conjunction ratio |  | -0.045 |  | 0.563 |  | -0.450 |  | -0.585 |  |
| Semantic VF |  | -0.901 |  | -2.008 |  | -0.802 |  | 0.979 |  |
| Simple sentence ratio |  | -0.837 |  | -0.017 |  | 0.414 |  | -0.193 |  |
| Syntactic complexity |  | -0.362 |  | 0.275 |  | 0.149 |  | 1.613 |  |
| Syntactic diversity |  | 0.241 |  | 0.536 |  | -0.176 |  | 0.998 |  |
| Type-token-ratio |  | 0.811 |  | 0.552 |  | 0.739 |  | 0.275 |  |
| Verb ratio |  | -0.330 |  | 0.763 |  | 0.979 |  | -1.600 |  |
| Verbal episodic memory (VLMT) |  | 1.255 |  | -1.779 |  | -0.370 |  | 1.258 |  |
|  | | | | | | | | | |

Note: *Betweenness* = measures the extent to which a node lies on the shortest paths between others; *closeness* = reflects how quickly information spreads from a given node to others; *strength* = indicates the overall level of connectivity/influence a node has in the network; *expected influence* = combines edge weights and network structure to estimate a node’s potential impact on the network.

**eTable 8.** Results of Network Comparison Test (NCT) with and without psychopathological variables.

| **NETWORK INVARIANCE TEST**  Test statistic M: 0  *p*-value 1 |
| --- |
| **GLOBAL STRENGTH INVARIANCE TEST**  Global strength per group: 23.03608 (with psychopathology), 23.03608 (without psychopathology)  Test statistic S: 0 |
| *p*-value 1 |
